# Highlight: Mitochondrial Mechanisms in Septic Cardiomyopathy

**DOI:** 10.3390/ijms160820095

**Published:** 2015-08-25

**Authors:** International Journal of Molecular Sciences Editorial Office

**Affiliations:** MDPI AG, Klybeckstrasse 64, 4057 Basel, Switzerland; E-Mail: ijms@mdpi.com; Tel.: +41-61-683-7734; Fax: +41-61-302-8918

## 1. Introduction

The paper “Mitochondrial Mechanisms in Septic Cardiomyopathy” [1], published in the current issue offers an excellent overview for readers of the *International Journal of Molecular Sciences*. We have asked several experts in the field about the significance and outlook of this paper. Following this, you will find a brief summary from the authors explaining the paper in their own words.

### 1.1. Dr. Jaime M. Ross and Dr. Giuseppe Coppotelli, Karolinska Institutet, Sweden

The review article, “Mitochondrial Mechanisms in Septic Cardiomyopathy” by Cimolai and colleagues, examines the role that mitochondria may play in the pathogenesis of cardiac dysfunction, a well-described, life-threatening complication in patients with severe sepsis and septic shock. Despite current therapies that mostly aim to reduce systemic infection and inflammation, sepsis remains the biggest risk factor for mortality among patients in critical condition. The authors elegantly discuss several possible mitochondrial mechanisms underlying septic cardiomyopathy and present studies that strongly support impaired energetics *per se*, rather than inflammation, as essential for treating cardiac dysfunction in order to improve outcome in septic patients.

### 1.2. Prof. Dr. Peter A. Ward, University of Michigan Medical School, USA

The paper by Cimolai *et al.* describes the biochemical pathways in mitochondria that become deranged during sepsis. These changes in mitochondria in the heart ultimately lead to depressed ATP synthesis which can rapidly result in cardiac failure involving both systolic and diastolic function. The earlier literature suggested that the heart failure of sepsis could be due to ischemia-reperfusion injury involving numerous organs (including the heart). As the authors point out, this has been disproven since arterial pO_2_ levels in the cardiac vasculature were not reduced during sepsis. Under a variety of conditions causing tissue damage, mitochondrial DNA (mDNA) is released, which can function as a “danger-associated molecular pattern” (DAMP) triggering a local and a systemic inflammatory response which may lead to multiorgan damage [2]. In sepsis, reactive oxygen species (ROS) (such as superoxide) and nitric oxide suppress mitochondrial function, ultimately leading to increased mitochondrial mass (swelling due to edema within mitochondria), which is often associated with mitochondrial dysfunction.

Our recent studies have highlighted an important role for extracellular histones in the setting of polymicrobial sepsis in rodents [3]. These histones appear to arise in a complement (C5a)-dependent manner related to activation of neutrophils, resulting in neutrophil extracellular traps (NETs) [4]. Extracellular histones are released in such circumstances. Histones *in vitro* can cause the following changes in cardiomyocyte mitochondria: binding of annexin V which correlates with apoptosis, reduced mitochondrial membrane potential, reduced levels of ATP, and increased mitochondrial mass (edema) [4]. Recent data have suggested that various interventions may reverse mitochondrial dysfunction (reviewed [1]). This may suggest interventions to “resuscitate” functionally defective mitochondria in the setting of sepsis and in other conditions.

### 1.3. Dr. Glòria Garrabou, University of Barcelona, Spain

Cardiac muscle is one of the most energetic demanding tissues of the body. It is full of mitochondria responsible of providing the ATP required for muscle contraction and heartbeat. Cells of septic patients seem to be unable to maintain intermediate metabolism and, consequently, develop an energetic failure. Mitochondrial dysfunction has been suggested as a potential cause of such energetic disarrangement which may be underlying septic cardiomyopathy.

The review by Cimolai *et al.* constitutes an outstanding summary and update of the experimental approaches performed in the last years to assess mitochondrial involvement in septic cardiomyopathy and potential mitochondrial therapeutics. Reviews were lacking in this topic and any advance in the field may help in saving lives worldwide.

The authors have performed a brilliant revision of the available literature and have the meritorious quality to make all the findings easy to understand to a non-specialized reader; helpful illustrations are also provided.

Congratulations again for publishing this work and thank you for taking into account my opinion.

### 1.4. Dr. Gabor Csanyi, Georgia Regents University, USA

Overall, this is a very informative review article by Cimolai *et al.* It is a well written study summarizing current knowledge in mitochondrial mechanisms leading to cardiac dysfunction in sepsis.

Although, the authors describe increased mitochondrial NO and ROS generation as potential players in sepsis, the precise molecular mechanisms by which these species contribute to mitochondrial dysfunction and septic cardiomyopathy remain unclear. For example, what are the protein, lipid, and DNA targets of NO, ROS, ONOO- during the development of cardiomyopathy in sepsis, and how nitrosylation and oxidative modification of these molecules lead to mitochondrial dysfunction?

The authors state that mitochondria are the most important source of ROS. It is a highly biased statement which has not been supported by references. Although, it is clearly an important source, there are other sources of ROS in the cell that could potentially contribute to cardiac dysfunction in sepsis. In fact, crosstalk mechanisms between mitochondrial and other ROS-generating enzymes should have been described in the article. There should have been a more extensive discussion of antioxidants in animal models of sepsis and clinical supplementation trials.

Mitochondrial biogenesis and mitophagy sections are well described.

## 2. A Summary of “Mitochondrial Mechanisms in Septic Cardiomyopathy”

**Figure:**
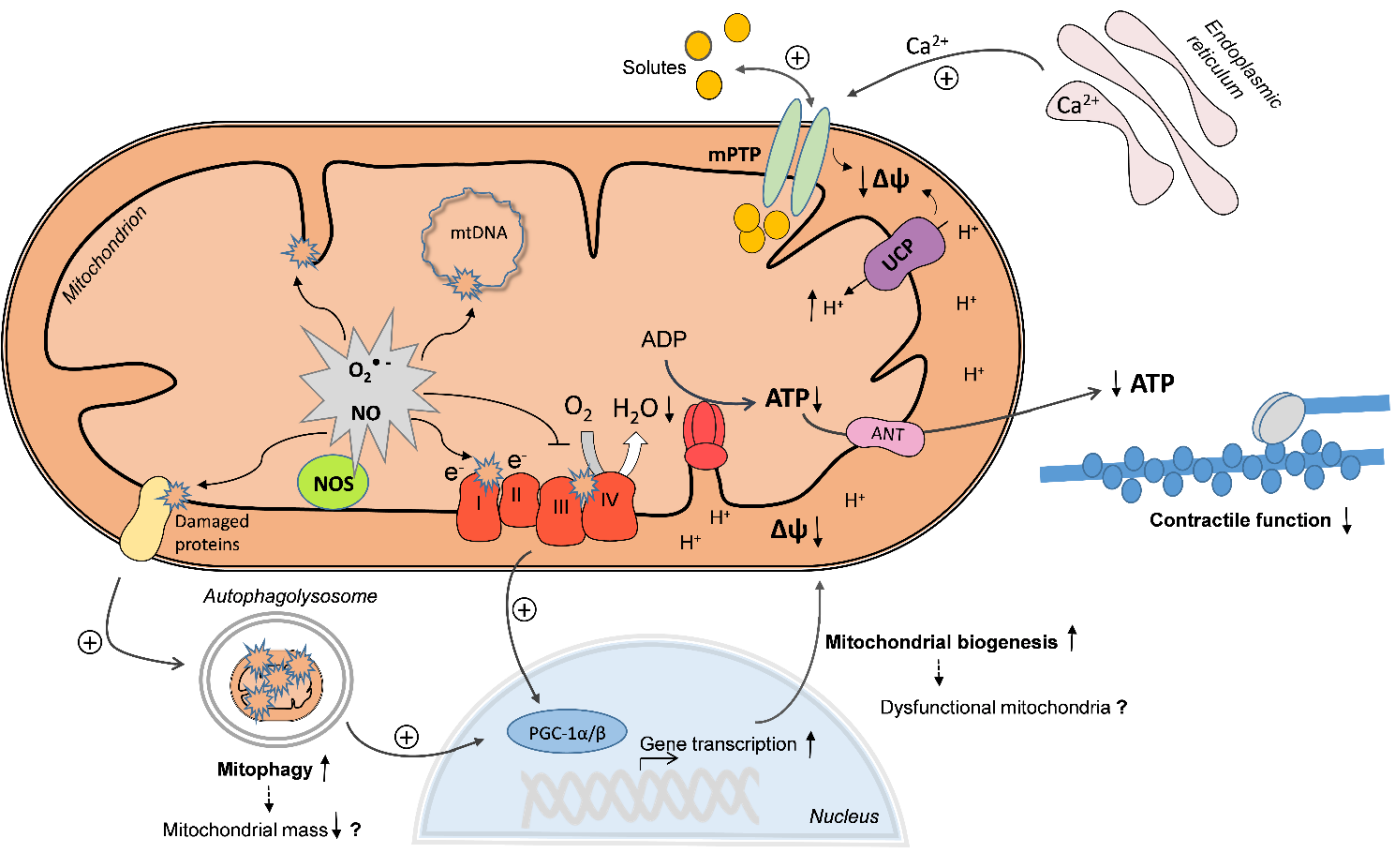


The Authors Explain:

Sepsis is a heterogeneous and dynamic syndrome caused by imbalances in the inflammatory network and nowadays still represents a major challenge in medicine due to the complexity of the disease, its rapid progression, the heterogeneity of the patient population and the lack of organ-specific treatment. Besides neutralizing the pathogen with antibiotics, the currently recommended therapeutic strategies are symptomatic, and mortality rates remain unacceptably high. The search for a specific treatment to restore organ dysfunction began with the discovery of additional pathophysiological mechanisms other than the already accepted ones. In this context, several decades ago, it was recognized that tissue hypoperfusion may lead to tissue hypoxia during sepsis, thereby contributing to impaired ATP generation. However, it was demonstrated later that O_2_ delivery to some tissues was not impaired or even improved during sepsis. Recent studies strongly suggest that organ dysfunction in sepsis is rather related to an impairment in cellular O_2_ utilization leading to cellular energy depletion, thus shifting the attention to mitochondrial function.

End-organ damage and organ failure in sepsis affects the most significant organs of the body, including the heart. Myocardial dysfunction is a well-described complication of severe sepsis, also referred to as septic cardiomyopathy, which includes both systolic and diastolic dysfunction. The occurrence of septic cardiomyopathy can increase the mortality rate up to 70% and is considered one of the major predictors of mortality. It was widely reported that decreased cardiac contractility correlated with impaired cardiac mitochondrial function in several animal models of sepsis. Some authors also reported alterations in myocardial mitochondrial structure in septic humans. Evidence of mitochondrial dysfunction in septic or endotoxemic animals includes decreased rates of State 3 respiration and ATP synthesis, decreased respiratory control ratios and membrane potential, decreased activities or expression of mitochondrial oxidative phosphorylation (OXPHOS) complexes, increased rates of State 4 respiration, increased structural alterations, and/or increased reactive oxygen species (ROS) production. However, the underlying mechanisms of cardiac mitochondrial dysfunction still remain incompletely understood. In the current issue of the International Journal of Molecular Sciences, we reviewed the publications of the last 15 years reporting mitochondrial alterations in the heart of septic or endotoxemic animals. As shown on the cover figure, the existing evidence suggests that diverse pathways can converge in the mitochondria, leading to dysfunction. Increased mitochondrial superoxide (O_2_^•−^) and nitric oxide (NO) production can cause direct oxidative or nitrosative damage and inhibition of OXPHOS complexes, resulting in decreased O_2_ consumption and decreased mitochondrial membrane potential (Δψ). In addition, Δψ may drop due to increased uncoupling protein (UCP)-mediated proton leak, increased Ca^2+^-induced mitochondrial permeability transition pore (mPTP) opening and by direct oxidative damage of the inner mitochondrial membrane. On the other side, increased mitophagy may eliminate dysfunctional mitochondria, which may be replaced by increased mitochondrial biogenesis, mediated by activation of peroxisome proliferator-activated receptor γ coactivator 1α/β (PGC-1α/β). However, if uncoordinatedly activated, mitophagy and mitochondrial biogenesis may lead to decreased mitochondrial mass or to the production of new, but dysfunctional mitochondria. As a consequence, mitochondrial ATP regeneration is compromised and energy depletion may contribute to cardiac contractile dysfunction.

Taken together, myocardial mitochondria may be considered both as a source and a target of damaging mechanisms that evolve during increased energy demands in sepsis. Future studies should elucidate the order of appearance of the above mentioned mechanisms in order to identify the critical mechanisms which may serve as potential targets of pharmacological intervention. In addition, studies using pharmacological modulators to prevent or reverse specific mitochondrial mechanisms are needed to further clarify the importance of mitochondrial mechanisms in the pathophysiology of septic cardiomyopathy and to define a feasible therapeutic approach.

## 3. Other Publications in *International Journal of Molecular Sciences*

For further reading, we list some related Special Issues:

- “Oxidative Stress in Cardiovascular Disease”
- “Mitochondrial Dysfunction in Ageing and Diseases”
- “Pathogenesis of Cardiac Arrhythmias and Heart Failure”
- “Improvement of Cardiac Function in Heart Failure”
- “Oxidative Stress in Cardiovascular Disease 2015”
